# Comparing the biological activity and composition of Cerebrolysin with other peptide preparations

**DOI:** 10.25122/jml-2024-0129

**Published:** 2024-01

**Authors:** Lisa-Franziska Seidl, Ludwig Aigner

**Affiliations:** 1 Institute of Molecular Regenerative Medicine, Paracelsus Medical University, Salzburg, Austria

**Keywords:** bioassay, Cerebrolysin, cerebroprotein hydrolysate, neuronal differentiation, porcine brain, DALYs, Disability-Adjusted Life Years, EGFP, Enhanced Green Fluorescent Protein, HPLC, High Performance Liquid Chromatography, NF-L, Neurofilament-L, PC12, Rat Pheochromocytoma Cell Line

## Abstract

Neurological disorders, ranging from acute forms such as stroke and traumatic brain injury to neurodegenerative diseases like dementia, are the leading cause of disability-adjusted life years (DALYs) worldwide. A promising approach to address these conditions and promote nervous system regeneration is the use of the neuropeptide preparation Cerebrolysin, which has been shown to be effective in both clinical and preclinical studies. Despite claims of similar clinical efficacy and safety by several peptide preparations, concerns regarding their generic composition and efficacy have been previously raised. Based on these reports, we analyzed the peptide composition and neurotrophic activity of several peptide preparations allegedly similar to Cerebrolysin and approved in some countries for treating neurological diseases. Our results demonstrate that these preparations lack relevant biological activity and that the peptide composition is significantly different from Cerebrolysin. peptide

## INTRODUCTION

Neurological disorders comprise the leading cause of disability-adjusted life years (DALYs) lost due to death or disability worldwide [1]. This highlights the urgent need for effective treatment options to diminish this immense burden of disease. Neurotrophic factors, a family of growth factors crucial for the development, function, and survival of adult neurons, have been extensively studied as a potential treatment option [2]. These factors are released in response to acute neurological damage or neurodegenerative diseases to protect and regenerate the affected neurons [3]. However, the use of neurotrophic factors in treating neurological disorders has not been successful since they cannot cross the blood-brain barrier or show considerable side effects [4].

Another approach that has been already explored for several decades is the use of neuropeptides that mimic the action of endogenous neurotrophic factors while being able to cross the blood-brain barrier. Neuropeptides are important in all stages of life, ranging from brain development to maintenance of neuronal homeostasis. Thus, it is not surprising that an imbalance of neuropeptides and neurotrophic factors is also a hallmark of neurological disorders. Therefore, the restoration of neuropeptide homeostasis is a promising approach in neuropathy therapy [5]. However, treatments using a single, specific neuropeptide tend to offer limited efficacy, as they may only address one aspect of the disorder, such as neuroprotection. Neurological disorders manifest as pleiotropic pathological conditions that need to be addressed by a multimodal approach that promotes not only neuroprotection but also neurogenesis and neuroplasticity. Thus, a promising strategy is the use of multimodal neuropeptide preparations. Cerebrolysin constitutes the originator product of multimodal neuropeptide preparations with a longstanding record of clinical efficacy and safety in various cerebrovascular and neurodegenerative diseases.

Several clinical trials have investigated the therapeutic potential of Cerebrolysin and demonstrated beneficial effects on neurorecovery across various neurological pathologies. Patients with acute ischemic stroke experienced significant improvements in neurological and functional outcomes following Cerebrolysin treatment [6]. Furthermore, Cerebrolysin treatment in conjunction with a structured neurorehabilitation program have led to substantial improvements in upper limb motor function and neurological outcomes [7,8]. Cerebrolysin also improved cognitive function and functional and rehabilitation outcomes in patients with moderate to severe brain trauma [9-11]. Importantly, clinical studies have not raised any safety concerns regarding Cerebrolysin, and a meta-analysis has shown that the occurrence of adverse effects after Cerebrolysin treatment is rare and comparable to that seen with placebo treatments [12].

Recent studies suggest that neuropeptide preparations that are allegedly produced in a similar way have distinct peptide profiles that can have a negative impact on their biological efficacy. Teng *et al*. [13] and Zhang *et al*. [14] demonstrated variability in efficacy both in vitro (cerebral endothelial cell permeability) and in vivo (in a rat model of embolic stroke) between Cerebrolysin and other peptide preparations. Teng *et al*. [13] already compared the composition of Cerebrolysin and cerebroprotein hydrolysate regarding their peptide fingerprints and could show profound differences in composition and biological activity.

Our aim was to systematically investigate a broad range of neuropeptide preparations from distinct manufacturers, including a deproteinized calf blood extract (Aktoseril), to assess their composition and neurotrophic activity. Cerebrolysin is known to induce neuronal differentiation, as evidenced by the expression of the neuronal cytoskeletal protein Neurofilament-L (NF-L) in the rat pheochromocytoma cell line PC12 [15]. Here, we used this system to compare the biological activity of various peptide preparations with the originator product Cerebrolysin.

## MATERIAL AND METHODS

### Material

Aktoseril (BIH Pharmaceuticals), Cebonin (Nexpharm), Cerabin-C (Unimed Pharmaceuticals), Cerebrain (Daewoong Pharmaceuticals), Cerebrin (B-Pharm), Cerebrolysin and an amino acid solution reflecting the amino acid component of Cerebrolysin (EVER Pharma), Cerebromine (Guju Pharm), Cerebropept (Kursk Biofactory Company 'BIOK'), cerebroprotein hydrolysate (Hangzhou Huajin Pharmaceuticals), Neurovera (Hyundai Pharm), Newrolizine (Huons), Solesejin (Kunil).

### Bioassay for neurotrophic activity

The materials and methods for cell cultivation and cell-based assay to evaluate the neurotrophic activity of the peptide preparations were used and performed as described by Seidl *et al*. [15]. Briefly, the enhanced green fluorescent protein (EGFP)-NF-L reporter PC12 cells were treated with 100 µl/ml of the indicated compounds for 4 days, followed by measurement of their EGFP mean fluorescence intensity using flow cytometry (BD FACSLyric). The Neurofilament-L Bioassay was validated for use with Cerebrolysin; thus, the results are stated as 'relative potency [%]', meaning percentage EGFP mean fluorescence intensity relative to a designated Cerebrolysin reference batch.

### High-Performance Liquid Chromatography analysis

The peptide fingerprint was obtained via reversed-phase High-Performance Liquid Chromatography (HPLC), as described by Teng *et al*. [13]. Chromatograms were generated using Agilent OpenLab CDS software.

### Statistical analysis

Cell-based assay data are shown as scatter plots with mean using GraphPad Prism 7 (version 7.04).

## RESULTS

### Literature

We conducted a literature search for published data already available for the compounds used in this study. Our search in PubMed for 'Cerebrolysin' returned 570 publications [16], indicating a substantial volume of research on this compound, whereas 'cerebroprotein hydrolysate' yielded 17 publications [17]. Searches for the remaining compounds returned no results. In addition, the Cochrane Library listed 270 trials related to Cerebrolysin and 9 trials for cerebroprotein hydrolysate, but none for the other preparations investigated in this work [18].

### Cerebrolysin is the only compound able to induce EGFP-NF-L expression

**Figure 1:**
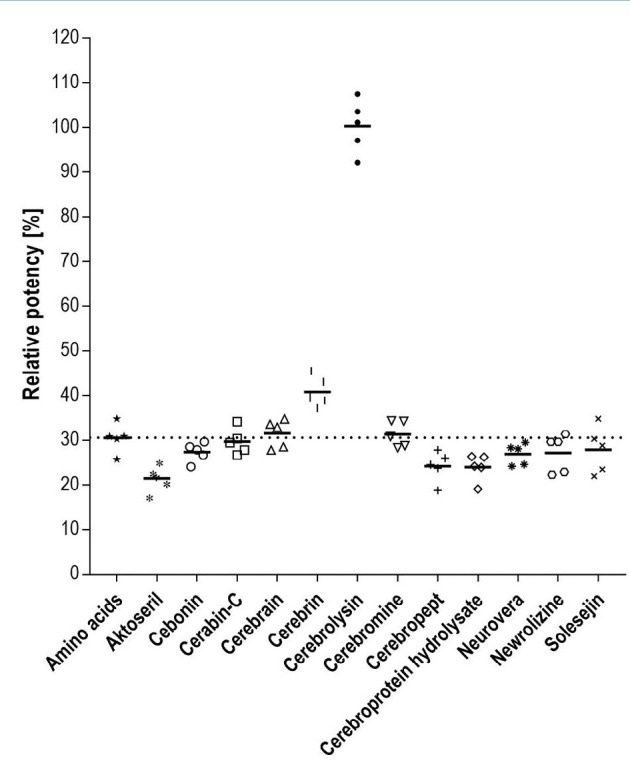
Cerebrolysin is the only compound able to induce relevant levels of EGFP-Neurofilament-L expression. Scatter plot with mean of Neurofilament-L Bioassay data from cells treated for 4 days with 100 µl/ml of the indicated compounds, 'amino acids' refers to the amino acid component of Cerebrolysin, n = 5. Dotted line shows the baseline neurotrophic activity represented by the median of 25 independent measurements of the amino acid component of Cerebrolysin.

As shown previously, Cerebrolysin induces the expression of Neurofilament-L. Subsequently, we investigated the NF-L-inducing capacity of other preparations marketed for treating neurological disorders. One batch of Aktoseril, Cebonin, Cerabin-C, Cerebrain, Cerebrin, Cerebrolysin, Cerebromine, Cerebropept, cerebroprotein hydrolysate, Neurovera, Newrolizine and Solesejin, respectively was tested against a Cerebrolysin reference. Except for Cerebrolysin, none of the products was able to induce NF-L expression that would be considerably above that of the amino acid solution used as a control (Figure 1).

### The peptide composition of preparations varies

**Figure 2:**
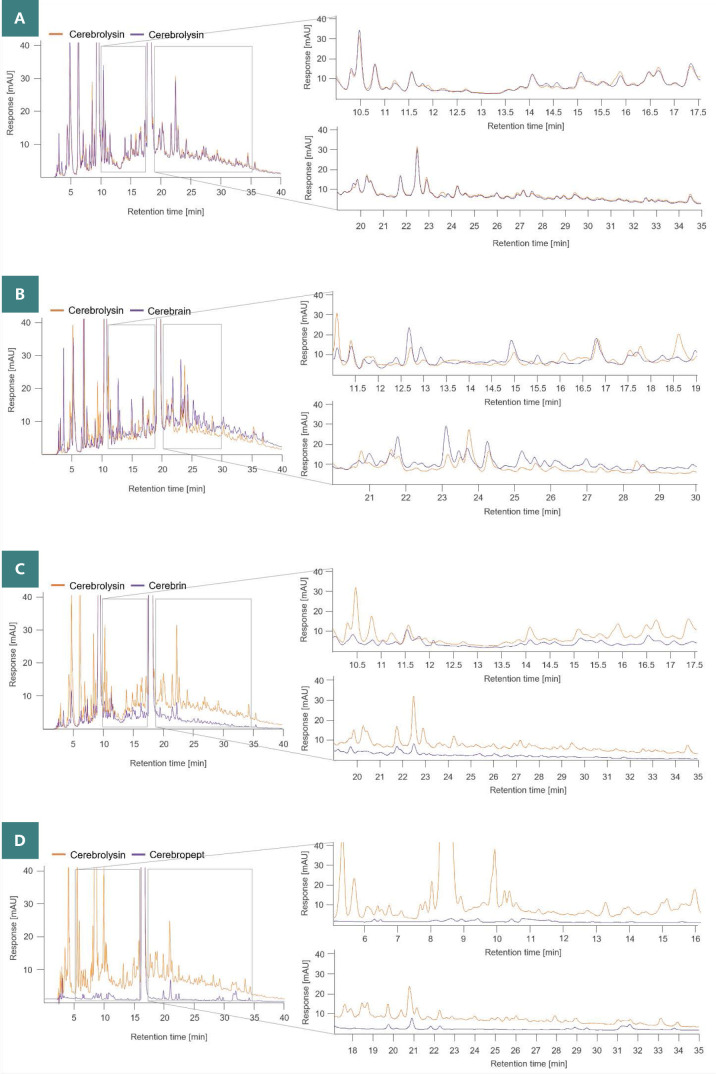
The peptide composition of preparations varies. Representative overlays of peptide fingerprint chromatograms, with sections of clear difference in the peak pattern and intensity enlarged. Overlay of (A) two different Cerebrolysin batches, as well as overlays of Cerebrolysin with (B) Cerebrain, (C) Cerebrin and (D) Cerebropept.

Teng *et al*. [13] previously demonstrated differences in peptide fingerprint between cerebroprotein hydrolysate and Cerebrolysin. Cerebroprotein hydrolysate is the common active ingredient of all the tested peptide preparations except Aktoseril, Cerebropept, and the originator product Cerebrolysin. Since the compounds could not induce NF-L expression like Cerebrolysin did, we assumed their peptide composition might also differ. To investigate this, we used reversed-phase HPLC to characterize the peptide profiles of the drugs. The resulting chromatograms indicated significant variations from Cerebrolysin, demonstrating the clear differences in the peptide composition of the preparations investigated (Figure 2A–D). Interestingly, even among different manufacturers using cerebroprotein hydrolysate as the common source of the active ingredient, the peptide composition of the compounds was also not comparable, suggesting different and non-standardized manufacturing steps for these products.

## DISCUSSION

Recent literature suggests differences in efficacy or composition between distinct neuropeptide preparations [13,14]. In this study, we examined these observations in 12 different compounds, which have received approval in some countries for treating acute or degenerative neurological diseases. All tested peptide preparations claim generic composition to Cerebrolysin and are approved with the same treatment regimen. Our findings revealed that, apart from Cerebrolysin, none of the tested compounds demonstrated neurotrophic activity and all of them had a significantly different composition when compared to Cerebrolysin. This investigation further reinforces that the biological activity and the peptide composition differ substantially among the examined preparations from the originator product Cerebrolysin. We could not find any clinical studies conducted with these compounds, so the data basis for their approval and clinical use is often unclear. Consequently, these various products can be expected to have a different clinical efficacy and safety profile from Cerebrolysin and, therefore, should not be considered interchangeable.

## CONCLUSION

Despite claims of similar clinical efficacy and safety by several peptide preparations, recent research suggests substantial differences in pharmacological activity and composition. We conducted a comprehensive study to investigate this topic and demonstrated that distinct peptide preparations differ substantially in composition and biological activity. The only compound found to have a profound clinical data basis and biological activity was Cerebrolysin.

## Data Availability

Further data is available from the corresponding author upon reasonable request.
